# Imaging the rapid yet transient accumulation of regulatory lipids, lipid kinases, and protein kinases during membrane fusion, at sites of exocytosis of MMP-9 in MCF-7 cells

**DOI:** 10.1186/s12944-020-01374-9

**Published:** 2020-08-23

**Authors:** Dominique C. Stephens, Tyrel W. Powell, Justin W. Taraska, Dinari A. Harris

**Affiliations:** 1 Department of Chemistry, Howard University, 525 College Street NW, Washington, D.C, 20059 USA; 2 Laboratory of Molecular Biophysics, National Heart, Lung, and Blood Institute, National Institutes of Health, Bethesda, MD 20892 USA

**Keywords:** Regulated exocytosis, Fluorescence microscopy, PIP2-mediated signaling, Lipids, Cancer

## Abstract

**Background:**

The regulation of exocytosis is physiologically vital in cells and requires a variety of distinct proteins and lipids that facilitate efficient, fast, and timely release of secretory vesicle cargo. Growing evidence suggests that regulatory lipids act as important lipid signals and regulate various biological processes including exocytosis. Though functional roles of many of these regulatory lipids has been linked to exocytosis, the dynamic behavior of these lipids during membrane fusion at sites of exocytosis in cell culture remains unknown.

**Methods:**

Total internal reflection fluorescence microscopy (TIRF) was used to observe the spatial organization and temporal dynamics (i.e. spatial positioning and timing patterns) of several lipids, and accessory proteins, like lipid kinases and protein kinases, in the form of protein kinase C (PRKC) associated with sites of exocytosis of matrix metalloproteinase-9 (MMP-9) in living MCF-7 cancer cells.

**Results:**

Following stimulation with phorbol myristate acetate (PMA) to promote exocytosis, a transient accumulation of several distinct regulatory lipids, lipid kinases, and protein kinases at exocytic sites was observed. This transient accumulation centered at the time of membrane fusion is followed by a rapid diffusion away from the fusion sites. Additionally, the synthesis of these regulatory lipids, degradation of these lipids, and the downstream effectors activated by these lipids, are also achieved by the recruitment and accumulation of key enzymes at exocytic sites (during the moment of cargo release). This includes key enzymes like lipid kinases, protein kinases, and phospholipases that facilitate membrane fusion and exocytosis of MMP-9.

**Conclusions:**

This work suggests that these regulatory lipids and associated effector proteins are locally synthesized and/or recruited to sites of exocytosis, during membrane fusion and cargo release. More importantly, their enrichment at fusion sites serves as an important spatial and temporal organizing “element” defining individual exocytic sites.

## Background

Exocytosis is a fundamental behavior, ubiquitous across eukaryotes and a variety of cell types. During exocytosis, vesicles fuse with the plasma membrane and result in secretion of biomolecules (vesicle cargo) to the outside of cells. This important mode of cellular communication can affect a variety of physiological processes, including polarized growth and motility [1, 2], cancer progression [3–5], and diabetes [6]. As it relates to cancer, dysregulation of exocytosis has been observed in a variety of cancer types and generally results from defects in several essential protein interactions involved in the discrete steps of exocytosis. Cancer progression relies, in part, on exocytosis to secrete a variety of protein factors, including growth factors, cytokines, proteases, and exosomes for establishment of tumor growth. During cancer progression, up-regulation of trafficking and secretion of several proteolytic enzymes, known as matrix metalloproteinases (MMPs) are responsible for degrading the extracellular matrix (ECM).

Metastasis occurs through a series of sequential steps [7, 8], where the tumor migrates from the primary site to colonize distant (secondary) sites and represents the most common cause of cancer death. During metastasis, matrix-degrading proteins, MMPs, degrade the ECM and are secreted due to precisely organized intracellular cell signaling events (i.e. specific spatial and temporal patterning of signals involved in intracellular signal transduction) in cells. These intracellular signaling pathways, facilitate exocytosis of secretory vesicles containing cargo protein, like MMPs by delivering secretory vesicles containing these proteases to the plasma membrane for subsequent release. Therefore, MMPs play an important role in cancer progression by altering cell invasion, migration, metastasis, and tumorigenesis [7, 9–11]. Because of their important role in ECM degradation, MMPs have often been used as major biological markers of metastasis and acquisition of metastatic traits in cancer cells. Several studies indicate that increased expression of MMPs is associated with several aggressive forms of breast, melanoma, ovarian, and colorectal cancers [12–15]. Furthermore, this increased expression of several MMPs, including MMP-9, MMP-2, and MT1-MMP (i.e. MMP-14), are secreted by a variety of metastatic cancer cells to aid in ECM degradation [16, 17].

During exocytosis, cells use more than twenty-five different proteins and an unknown number of lipids [18] to result in the externalization of the secretory vesicle cargo molecules [19]. This is mediated by a number of signal transduction pathways, which facilitate a cascade of important protein-protein and lipid-protein interactions, and lipid signaling events in these cells [19]. The minimal machinery required for fusion is a complex of three proteins, syntaxins, synaptosomal-associated proteins (SNAPs), and vesicle-associated proteins (VAMPs), collectively called the soluble N-ethylmaleimide–sensitive factor attachment protein receptors (SNAREs) [18, 20, 21]. This complex of proteins coil together to pull the plasma membrane and vesicle membrane into close apposition to drive fusion [20].

Although the three SNARE proteins represent the minimal fusion machinery necessary for exocytosis, fusion mediated by SNAREs solely, is relatively slow and uncoordinated. Therefore, though the SNAREs induce membrane fusion in vitro, there are dozens of other accessory proteins and lipids that assemble together with the SNAREs to accelerate and regulate exocytosis in cells [18, 22]. The additional biomolecules essentially help to spatially and temporally coordinate the distinct steps associated with secretory vesicle fusion and exocytosis. Moreover, the SNAREs and the additional factors help to provide spatial and temporal cues or spatiotemporal organization associated with sites of secretory vesicle fusion (i.e. exocytic sites). The role of spatial and temporal organization at exocytic sites has been generally shown to occur through membrane specialization or organized spatial regions. These organized and coordinated regions in the cells have elevated levels of these factors at exocytic sites [23]. Specifically, the SNAREs, accessory proteins and lipids form distinct organizing “elements” near the cell membrane. These organizing “elements” interact with components of secretory vesicles and help facilitate exocytosis in cells. Fundamentally, these organizing “elements” can be grouped into three distinct classes and include: (1) specialized scaffolding proteins; (2) specialized lipids; and (3) the actin cytoskeleton network proteins [23].

The role of specialized lipids has become of increasing importance and overwhelming evidence suggests that specialized lipids, including regulatory lipids and other bioactive lipids, act as lipid-signaling mediators and affect a variety of cellular functions (e.g. signaling and regulation) [24]. One known cellular function involving specialized lipids is exocytosis and the sequential stages underlying exocytosis, including secretory vesicle trafficking, docking, priming, vesicle fusion, and recapture [19]. The most notable of specialized lipids is the regulatory lipid, phosphatidylinositol-4,5-biphosphate (PI4,5P or PIP2) [25–27]F148, [28, 29], which is: (1) a prerequisite for Ca^2+^-dependent exocytosis [30]; (2) coordinates trafficking of secretory vesicles to their docking sites on the plasma membrane [31]; and (3) primes secretory vesicles for exocytosis, by recruiting accessory proteins and interacting with key components of the exocytic machinery (e.g. SNARE proteins) [32]. Additionally, the major upstream lipid precursor of PIP2 synthesis, phosphatidylinositol 4-phosphate (PI4P or PIP) is another distinct phosphoinositide (PI) lipid species, which regulates exocytosis by promoting vesicle docking [33]. While the downstream degradation product of PIP2 hydrolysis, diacylglycerol (DAG) promotes exocytosis by promoting vesicle recruiting through the mammalian uncoordinated protein, Munc13–1, a vesicle priming protein [34].

The regulatory lipid, DAG has two possible fates, either phosphorylation to become the bioactive lipid, phosphatic acid (PA) or activation of protein kinase C (PRKC) via allosteric modification. Both downstream cellular fates have been functionally linked to exocytosis. PA can recruit proteins, like the SNAREs, thereby facilitating priming and vesicle fusion [35–37]. PRKCs are critical regulators of exocytosis through phosphorylation of distinct protein targets effecting or regulating components of the exocytic machinery, like Munc18 or SNAP-25 [38–43]. The interconversion of these various distinct specialized lipids, as well as, downstream activation by DAG is achieved by specific lipid kinases, phospholipases, phosphatases, and protein kinases [44, 45]. Furthermore, these effector proteins have also been found to promote distinct *steps* involved in exocytosis [46–48]. Therefore, it suggests that local synthesis and degradation of these specific lipid species is achieved through the specific accumulation of these effector proteins at sites of membrane fusion and exocytosis [23, 26, 44, 49–51]. Overall, this first implies that PIP2-mediated signaling is an important signal transduction pathway, involving a cascade of specialized lipids, and a variety of effector proteins, that are functionally linked to exocytosis. And secondly, these specialized lipids presumably accumulate in defined microdomain regions, with some spatial and temporal patterning, in order to recruit effector proteins and promote exocytosis.

More and more, total internal reflection fluorescence (TIRF) has been utilized to image the spatiotemporal organization and dynamics associated with exocytosis in a variety of cellular contexts. This includes: (1) exocytosis involved in neurite elongation [52]; (2) exocytosis associated with cytoskeleton rearrangements and the formation of membrane fusion “hotspots” [53]; (3) viral exocytosis [54]; (4) microvesicles exocytosis [55]; (5) dense core vesicle (DCV) exocytosis [56] and; (6) secretory vesicle exocytosis [57]. Previously it was shown using two-color TIRF that it was possible to study the spatiotemporal patterning and dynamic behavior of several red fluorescently-labeled Rab GTPases, Rab effector proteins, and SNARE proteins (organizing element #1; specialized scaffolding proteins) during regulated exocytosis of MMP-9, at individual membrane fusion sites or exocytic sites in MCF-7 adenocarcinoma cancer cells [57].

Here, to visualize exocytic vesicle fusion, again two-color TIRF microscopy was exploited to image the spatial organization and temporal dynamics associated with several specialized lipids (organizing “element” #2) and effector proteins at exocytic sites of MMP-9 from MCF-7 cells. To this end, red fluorescent protein-tagged lipid-binding sensor proteins, with known specificity for single and distinct lipids [58–62], were used to monitor the dynamics of several specialized lipids at exocytic sites of green fluorescent protein-tagged MMP-9 (MMP9-GFP). After stimulating exocytosis, with the known tumor promotor drug, phorbol myristate acetate (PMA), a transient accumulation of the regulatory lipids, PIP2 and DAG, was observed at exocytic sites during membrane fusion. Also, a similar recruitment of distinct lipid kinases and protein kinases (i.e. PRKCs) was observed at these exocytic fusion sites. This approach has allowed the systematic mapping of the dynamic behavior of lipids and proteins associated with PIP2-mediated signaling, during exocytosis of MMP-9 in MCF-7 cells.

## Material and methods

### Plasmids

Most plasmids were obtained fused to fluorescent proteins and when necessary the fluorescent protein was replaced specifically with the monomeric red-fluorescent protein, mCherry in order to conduct two-color TIRF. In general, this was done by double restriction digest with the *BsrG-1*, a common unique sequence located near the downstream 3′ end in the most fluorescent protein sequences and a different sequence associated with upstream 5′ end multiple cloning sites in the plasmid. The fluorescent sequence was removed from the acceptor plasmid, while the red-fluorescent (mCherry) sequence was inserted and ligated from the donor plasmid, into the acceptor plasmid. Otherwise fusions were constructed from plasmids using either mCherry-N1 or mCherry-C1 directly from the unfused plasmid. MMP9-GFP (pEGFP-N1-MMP9) was a gift from Rene Harrison (University of Toronto, Toronto, ON). *Lipid sensors.* PIP2-sensor (GFP-C1-PLCdelta-PH, addgene #21179) [62]; DAG-sensor (GFP-N2-PRKCdelta-C1, addgene #21216) [61]; PIP-sensor ((PIP^P4M^; mCherry-P4M-SidM; addgene #51471) [63]; PIP-sensor ((PIP^Osh^), pRS406-PHO5-GFP-2xOsh2 PH domain (short), addgene #58829); PA-sensor (Raf1-mCherry generated from pDONR223-Raf1, addgene #23832) [64]; farnesylated-anchor (mCherry-Farnesyl-5, addgene #5505. *Lipid kinases.* PI4K3A (mTq2-PI4K3A), PI4K3B (GFP-PI4K3B), PI4K2A (PI4K2A-GFP), and PI4K2B (PI4K2B-GFP) were gifts from T. Balla, National Institutes of Health, Bethesda, MD) [63]; PIP5KA (mCherry-PIP5Kα) and PIP5KB (mCherry-PIP5Kβ) were gifts from S. Schmid, UT Southwestern Medical Center, Dallas, TX) [65]; PIP5KG (mCherry-PIP5K1c generated from DNASU: HsCD00000979). *Protein kinases.* PRKCA (PRKCalpha-mCherry generated from PRKCalpha WT, addgene #211232), PRKCB (PRKCbeta-mCherry generated from PRKCbeta II, human), PRKCG (PRKCgamma-mCherry generated from pENTR-PRKCG, addgene #16180), PRKCD (PRKCdelta-mCherry generated from PRKCdelta WT, addgene #16386), PRKCE (PRKCepsilon-mCherry generated from FLAG-PRKCepsilon, addgene #10795), and PRKCZ (PRKCzeta-mCherry) were gifts from J. Taraska, National Institute of Health, Bethesda, MD); *Rab27 GTPases and prenylation mutants.* GFP-C1-Rab27a, GFP-C1-Rab27b, GFP-C1-Rab27a (GER), and GFP-C1-Rab27b (GER) were gifts from W. Westbroek, National Institute of Health, Bethesda, MD).

### Cell culture and transfection

MCF-7 cell stocks were maintained as previously described [57]. Using coverslips coated with collagen, MCF-7 cells were plated and transfected using Lipofectamine 2000 (Invitrogen) and 1 μg of each plasmid. Following transfections, cells were imaged in imaging buffer (in mM: 10 glucose, 10 HEPES, 130 NaCl, 2.8 KCl, 5 CaCl_2_, and 1 MgCl_2_) at pH 7.4. Prior to imaging, exocytosis was induced using 500 nM PMA for 30 min, after serum-starving transfected cells for 1 h.

### TIRF microscopy

The details on TIRF microscopy were described previously [57]. Briefly, using an inverted fluorescent microscope (IX-81; Olympus) with a 100X/1.45NA objective (Olympus), cells were excited using a combination of green (488 nm) and red (561 nm) lasers, passed through a LF405/488/561/635 dichroic mirror, and filtered emitted light was projected side-by-side on an electron multiplying charge-coupled device (EM-CCD) camera (DU 897; Andor). Using Andor IQ2 software, images of transfected cells were obtained at 5 *Hz* and 100 ms exposure times. Each day fluorescent beads (Invitrogen) were imaged in the green and red channels and superimposed by mapping corresponding fluorescent bead positions in both channels. The green and red images were subsequently transformed and aligned as described before [56, 66]. All experiments were conducted at room temperature (^~^ 25 °C).

### Structured illumination microscopy (SIM)

TIRF-SIM imaging was conducted on a DeltaVision OMX SR structured illumination microscope. An inverted microscope was equipped with a spatial light modulator (SLM) that diffracts the beam using multiple grating patterns. In combination with a 100X/1.49NA oil immersion objective lens, 488 nm (GFP) and 561 nm (mCherry) excitation is passed through a multi-band dichroic mirror (DM4) and green/red images were superimposed during processing with OMX SR task builder.

### Image analysis

The details on image analysis was extensively described previously [57], using the same TIRF-based imagining technique to study organizing “element” #1 (scaffolding proteins). Briefly, using ImageJ (NIH) and custom Matlab scripts (Mathworks), for correlation analysis, local maxima of MMP9-GFP were identified in the green channel and centered in a small (4 μm × 4 μm) cropped region, before normalization and averaging. This was also done in the corresponding cropped regions in the red channel. The intensities in the red channel (and randomly selected regions) were determined using radial scans analysis on the cropped regions and were averaged for each frame. As detailed in [57], fluorescence intensities were background corrected, prior to calculating both average peak height and Pearson’s correlation coefficient (*c*) for each red-fluorescent labeled protein.

For exocytic fusion events analysis, fusion event coordinates and stack positions were identified by hand-selecting, as previously discussed [57]. All time-aligned fusion events (green channel) and corresponding regions in the red channel were centered in a cropped region (480 nm square box), background corrected, normalized from 0 (minimum) to 1 (maximum), and averaged to determine mean fluorescence intensity trajectories. This was done for every fusion event from − 10 s to + 50 s relative to the first frame of the fusion event. For spatial patterning analysis (i.e. correlation analysis) and temporal dynamics analysis (i.e. exocytic fusion event analysis), 15 cotransfected cells were imaged for experiments.

### Western blotting

For detection of phosphorylated Rab protein (Rabphilin), cells were treated with and without PMA simultaneously in separate culture flasks. Cells were harvested, pelleted, and lysed with CHAPS Stock Solution 0.25% [w/v] in PhosphoProtein Lysis Buffer, with 1 complete protease inhibitor and Benzonase® Nuclease. Following lysis, the mixture was kept on ice for 30 min, centrifuged (10,000 x g) at 4 °C for 30 min, and the supernatant was harvested, prior to determining the protein concentration. After adjusting the concentrations to 0.1 mg/ml, the protein lysates were added to the PhosphoProtein Purification Column. Using PhosphoProtein Elution buffer, the isolated phosphoproteins were collected, and concentrations determined. Conditioned media phospho lysate protein content was quantified, run on a protein gel (using 10 μg protein lysate), transferred to a polyvinylidene fluoride (PVDF) membrane, blocked for 1 h at room temperature, and incubated with a Rabphilin 3A (Ser234) antibody overnight at 4 °C. The following day the membrane was washed, incubated with a secondary antibody (anti-mouse, 1:2000), washed thoroughly again, before enhanced chemiluminescence treatment and exposure to film. The anti-phospho Rabphilin 3A (Ser234) antibody was purchased from PhosphoSolutions (#p1553–234).

## Results

### Systematic spatial mapping reveals weak or non-specific association of specialized lipids with docked secretory vesicles containing MMP-9

MCF-7 breast cancer cells, which can be used as a model system for regulated exocytosis of MMP-9, were used to probe the lipid environment of secretory vesicles containing MMP-9 and their subsequent exocytosis in living cells. To determine the spatial arrangement of lipids at MMP9-containing secretory vesicles near the plasma membrane, two-color TIRF microscopy was utilized to image the colocalization of these vesicles with various specialized lipids in living MCF-7 cells. For colocalization experiments and to probe the dynamic lipid environment at secretory vesicles and exocytic sites, the secretory vesicle marker, MMP9-GFP was used and cotransfected with a specific lipid sensor fused to the red fluorescent protein, mCherry. Specific lipid species are visualized with lipid sensors, which are fluorescently-tagged protein domains with known specificity for distinct lipid species. Lipid sensors are protein domains from lipid binding proteins, like pleckstrin homology (PH) domain from phospholipase C (PLC). These lipid sensors are well-characterized and have specific binding to a particular lipid species [58] and are routinely used to image the endogenous lipid distribution in living cells [61, 62]. Here, they were used to image the spatial organization and dynamic behavior of these specialized lipids at exocytic sites of MMP-9.

**Fig. 1:**
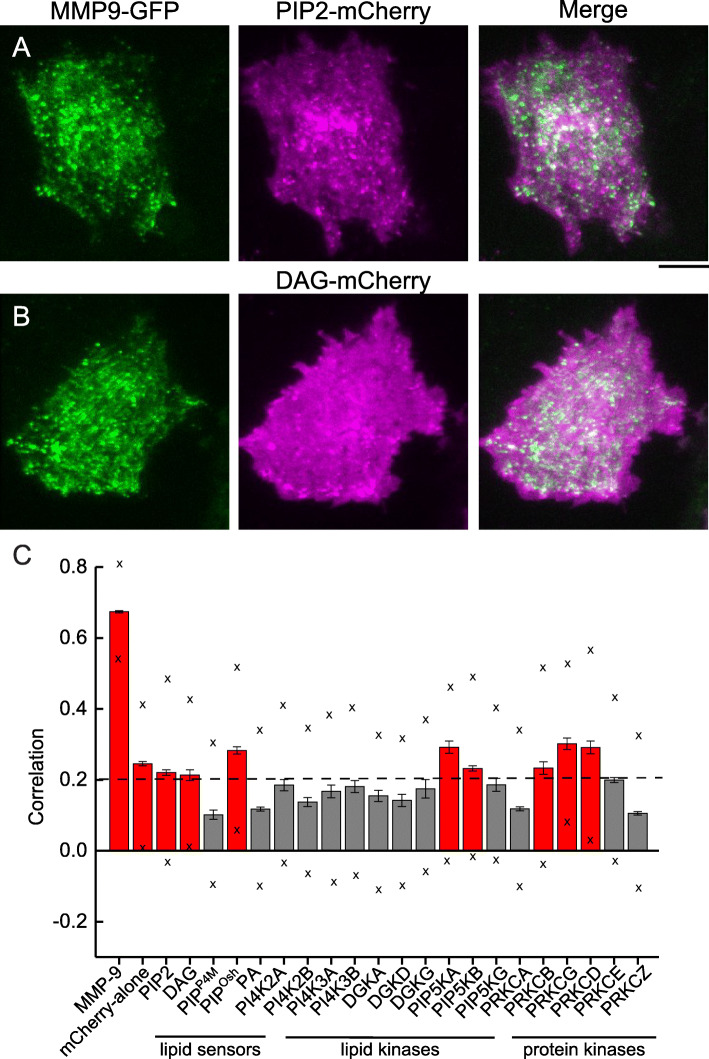
Correlation/ colocalization analysis of secretory vesicles containing MMP9-GFP with lipid sensors tagged with a red-fluorescent protein: **a** PIP2-sensor; or **b** DAG-sensor. TIRF images from green channel of MMP9-GFP (left); red channel of either PIP2-mCherry or DAG-mCherry lipid sensor (middle); and merged image (right) from an MCF-7 cell. Scale bar, 10 μm. **c** Correlation analysis of 23 proteins associated with MMP9-GFP–labeled secretory vesicles in MCF-7 cells, including lipid sensors, lipid kinase isoforms, and protein kinase C isoforms (PRKCs). Red boxes above 0.2 (referenced by the dotted line) indicate proteins associated with secretory vesicles, and boxes below 0.2 represent nonspecifically or not associated proteins. Standard error (SE) is depicted as whiskers and the standard deviation (SD) as × marks

A well-established TIRF-based imaging screen [55–57, 67] was used to image and subsequently determine colocalization (or correlation) following cotransfections. This unbiased image mapping approach allowed determination of the degree of association between the test, red-labeled protein and all visible MMP9-labeled vesicles and control images (Fig. 1). Imaging revealed that docked secretory vesicles containing MMP9-GFP were detected as punctate structures, while correlation analysis showed broad labeling and distribution of lipid sensors across the plasma membrane. None of the lipid sensors tested appeared to be solely or highly enriched at docked secretory vesicles containing MMP9-GFP. Figure 1 shows two separate lipid sensors tested, including the PIP2-sensor (Fig. 1a) and the DAG-sensor (Fig. 1b). The PIP2-sensor used is a fusion of the PH domain of phospholipase C δ1 (PLC-δ1) with the red-fluorescent protein, mCherry [62] and the DAG-sensor is a fusion of the C1 domain of PRKCδ with mCherry. Both the PIP2-sensor and the DAG-sensor recognize only one distinct specific lipid species and therefore represents suitable in vivo indicators of that lipid species. Similar to previous reports of these lipid sensors [61, 62], a heterogenous distribution of both lipid sensors was observed. Both lipid sensors show more ubiquitous expression across the plasma membrane and punctate localization associated with docked membrane-bound secretory vesicles in MCF-7 cells.

Correlation values for the red-labeled lipid sensors and associated effector proteins relative to secretory vesicles containing MMP9-GFP were separated into four categories (highly correlated, moderately correlated, weakly correlated, and non-specifically/not associated) [56, 57, 67]. In general, correlation values of red-labeled proteins above > 0.20 represent colocalized proteins (highly, moderately, and weakly correlated) and below < 0.20 represent correlation values of nonspecifically or not associated proteins, as was previously used with similar TIRF-based imaging techniques [56, 57]. The lipid sensors tested showed a range of low correlation values (*c* = 0.02–0.31) with MMP-9 (Fig. 1c) and do not appear to be statistically different than the relatively nonspecific cytosolic and membrane-anchored probes tested (controls; cytoplasmic-mCherry and farnesyl-mCherry). Though many secretory vesicles containing MMP9-GFP colocalized with the lipid sensors were observed, the correlation values are relatively low because of the overwhelming number of MMP9-GFP secretory vesicles that did not show punctate staining with the lipid-sensors. As controls, the highest correlated protein was associated with MMP9-mCherry (*c* = 0.67), while the correlation value for free cytoplasmic-mCherry (mCherry-alone) was 0.24. Both these values are consistent with the previous report [57]. Correlation values ranging from 0.20–0.34 represent weakly colocalized proteins and lipid sensors, and include PIP2, DAG, PIP^Osh2^, PIP5KA, PIP5KB, PRKCB, PRKCG and PRKCD, while values below 0.20, include PIP^P4M^, PA, PI4K2A, PI4K2B, PI4K3A, PI4K3B, DGKA, DGKD, DGKG, PIP5KG, PRKCA, PRKCE and PRKCZ (Fig. 1c). Collectively this represents several lipids associated with the PIP2-mediated signaling pathway, as well as several lipid kinase isoforms and protein kinase C isoforms that are responsible for the synthesis of these lipids or are downstream effectors of these lipids. Overall, this spatial patterning data suggests that the majority of specialized lipids, lipid kinases, and protein kinases are only weakly or nonspecifically/not associated with docked MMP9-containing secretory vesicles.

### Transient recruitment of regulatory lipids to exocytic sites

Next, the temporal dynamics associated with the lipid environment at exocytic sites was characterized during membrane fusion. To promote secretion of MMP-9, MCF-7 cells were stimulated using the tumor promotor, PMA [68], which reproducibly triggers robust exocytosis of MMP-9 in these cells. Using two-color TIRF, the behavior of red fluorescently-tagged lipid sensors (red channel) at fusion sites was imaged during PMA-stimulated exocytosis of MMP9-GFP (green channel). Exocytic sites or fusion events were identified as a sudden sharp increase (or spike) in MMP9-GFP from a docked secretory vesicle, followed by a rapid fluorescence decay as MMP-9 diffuses away from the site of exocytosis. This dynamic behavior observed using TIRF is a hallmark of exocytosis and has been observed at single sites of exocytosis, including the release of: (1) NPY from DCVs in INS-1 cells [56]; (2) vesicular acetylcholine transporter (VAChT) from small synaptic vesicle-like microvesicles (SLMV) in PC12 cells [55]; and (3) MMP-9 from secretory vesicles in MCF-7 cells [57]. By monitoring the decay kinetics, quantitating the fluorescence intensity changes (in both the green and red channels) at individual exocytic sites of MMP-9, and averaging fusions events from many MCF-7 cells, an average time-dependent fluorescence change from both channels was produced.

**Fig. 2:**
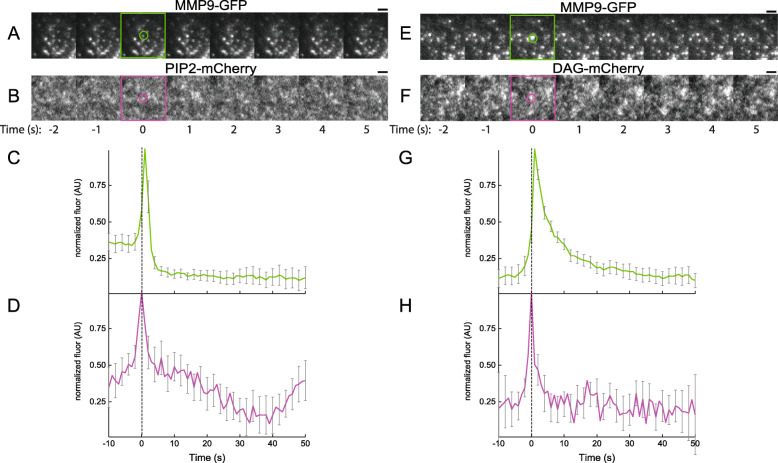
TIRF images of cotransfected MCF-7 cells containing MMP9-GFP and red fluorescent-labeled PIP2-sensor (**a**–**d**) or DAG-sensor (**e**–**h**). Exocytic fusion events analysis in the MMP9-GFP (green channel; **a**, **e**) and the corresponding area in the red channel with the PIP2-sensor (**b**) or DAG-sensor (**f**), in PMA-stimulated cells. Time-lapse snapshots (with timepoints) of fusion events are shown, along with average normalized fluorescence intensity trajectories in the green (**c** & **g**) and red channels (**d** & **h**). The SE of the data are plotted as gray lines. Number of fusion events (*N*) used in the averaging are *N* = 25 for PIP2-sensor and 33 for DAG2-sensor. Dashed vertical line corresponds to the fusion event (0 s) of MMP9-GFP in the green channel, which was time-aligned to the red channel

The temporal dynamics of regulatory lipids at exocytic sites of MMP-9 in MCF-7 cells was first examined. In regulated exocytosis, the importance of regulatory lipids, PIP2 and DAG, are well documented as secondary messengers involved in several cellular signaling pathways [49], including the distinct steps of regulated exocytosis [27, 30, 34, 43, 45, 50, 58, 61, 62, 69–71]. PIP2 is a distinct lipid species that is associated with recruiting and priming vesicles for exocytosis, via interactions with essential exocytic proteins [69]. DAG is another regulatory lipid implicated in controlling exocytosis and has been shown to increase stimulus-coupled secretion in neuroendocrine cells [34]. Because of the known roles and involvement of these regulatory lipids in exocytosis, the temporal dynamic fluorescence changes associated with either the PIP2-sensor or DAG-sensor (red channel) was imaged at sites of exocytosis of MMP9-GFP (green channel). Figure 2 shows representative time-aligned snapshots and average fluorescence decay trajectories from fusion sites of MMP9-GFP in the green channel (snapshots: Fig. 2a for PIP2-sensor and Fig. 2e for DAG-sensor; trajectories: Fig. 2c for PIP2-sensor and Fig. 2g for DAG-sensor), and the corresponding areas in the red channel for the PIP2-sensor (snapshots: Fig. 2b; trajectories: Fig. 2d) and DAG-sensor (snapshots: Fig. 2f; trajectories: Fig. 2h). It was found that both regulatory lipid sensors are transiently enriched at sites of exocytosis of MMP-9, at the moment of fusion before diffusing away from exocytic sites (Fig. 2d and h). Accumulation of both PIP2 and DAG at exocytic sites have broadly similar kinetics (i.e. sharp rise followed by slower decay). This suggests that enrichment of regulatory lipids occurs only during membrane fusion at individual exocytic sites of MMP-9. There was no observable accumulation of the farneslyated-mCherry (a lipid-anchored protein control), mCherry-alone protein control, or the mutated PIP2-sensor, which is unable to bind PIP2 [62], at fusion events (Supplemental Figure 1A-C).

Since DAG is generated directly from PIP2 hydrolysis by PLC, it suggests that the DAG might be locally synthesized at exocytic sites following recruitment and/or synthesis of PIP2 at these sites. Because the PIP2-sensor used contains the PH domains from PLC [62], it was predicted that exocytosis of MMP-9 would be altered in the presence of an inhibitor of PLC. Inhibition of PLC stops exocytosis of MMP-9 was observed, following the addition of 10 μM of U-73122, a PLC inhibitor. There were no observable fusion events and no vesicle trafficking between docked sites, in the presence of the PLC inhibitor. This suggests that PLC inhibition blocked local hydrolysis of PIP2 and DAG synthesis, and subsequent loss of MMP-9 release. It is worth noting that a non-specific effect could be caused by the inhibition and therefore cannot be discounted. Overall, the TIRF data suggest that regulatory lipids PIP2 and DAG, have dynamic temporal behaviors at exocytic sites of MMP-9 in MCF-7 cells. Additionally, it demonstrates that the specialized lipids are transiently accumulated at these spatial positions at precisely the moment of fusion.

### Accumulation of specialized lipids and lipid kinases to exocytic sites

Since the regulatory lipid, PIP2 is transiently enriched at exocytic sites during the release of MMP-9, the role of the upstream lipid and lipid kinases involved in the pathway of PIP2 synthesis was examined. PIP2 can be synthesized through the action of two distinct but related phosphoinositide kinases (i.e. lipid kinases) [72]. First, the phosphorylation of the lipid, phosphatidylinositol to phosphatidylinositol 4-phosphate, PI4P (or PIP), occurs and represents the first committed step in the generation of PIP2. This reaction is catalyzed by the lipid kinase, PI 4-kinase (PI4K). Second, the lipid, PI4P (or PIP) undergoes another phosphorylation to phosphatidylinositol-4,5-bisphosphate, PI45P (or PIP2), by the lipid kinase, phosphatidylinositol 4-phosphate 5-kinase (PIP5K).

**Fig. 3:**
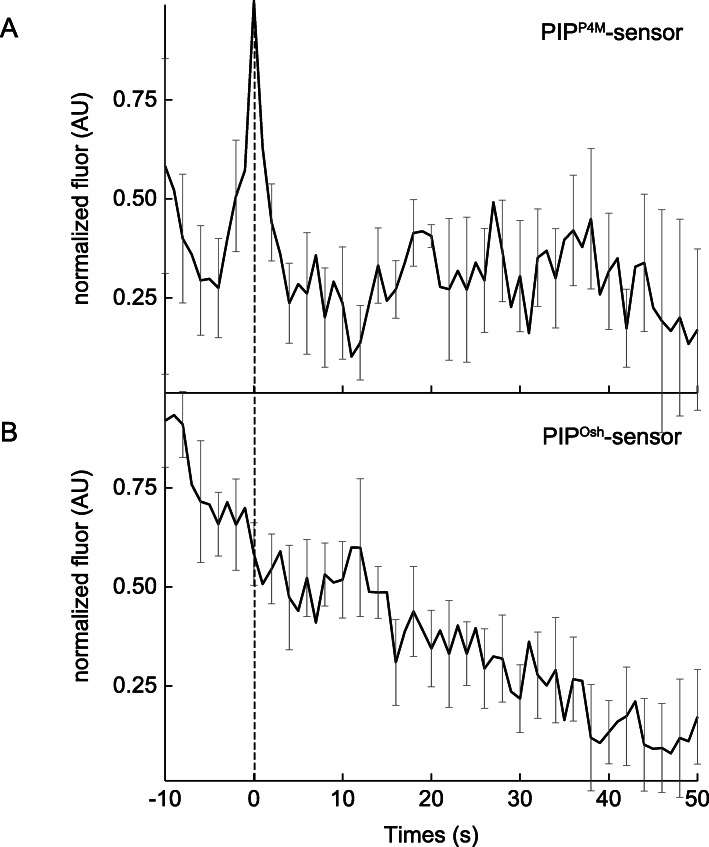
Average fluorescence intensity changes (dark line; background-subtracted and normalized) associated with the red fluorescent-labeled PI4P- or PIP-sensors, including: **a** PIP^P4M^, *N* = 15; and **b** PIP^Osh^, *N* = 19 and the SE (gray) for all traces. Dashed vertical line corresponds to the fusion event (0 s) of MMP9-GFP in the green channel, which was time-aligned to the red channel

The dynamics associated with two different PIP lipid sensors were tested, as well as four different PI4K isoforms at exocytic sites of MMP-9. Two different PIP lipid sensors were used to monitor dynamics during the exocytosis of MMP-9 from MCF-7 cells. One of the PIP lipid sensors was derived from the PI4P binding of the SidM (P4M) domain of the secreted effector protein SidM from the bacterial pathogen *Legionella pneumophila* [63]. A transient accumulation of PIP^P4M^ was observed around the time of fusion, followed by rapid diffusion decay away from the membrane upon vesicle cargo release (Fig. 3a). The second PI4P lipid sensor was derived from the PH domain from oxysterol (Osh) binding protein [73, 74] and can recognize PI4P, similar to P4M, but represents an older and far less sensitive probe for plasma membrane PI4P. Using this PIP lipid sensor, PIP^Osh^, a decrease in fluorescence over time following the exocytosis of MMP-9 (Fig. 3b) was observed. It is worth noting that these differing findings with the varying PIP lipid sensors are consistent with reports of different PI4P binding domains probing distinct cellular populations [63].

**Fig. 4:**
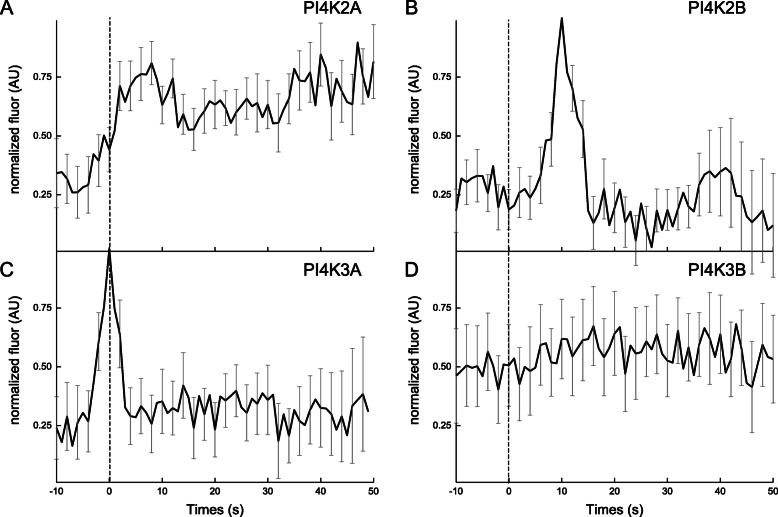
Average fluorescence intensity changes (dark line; background-subtracted and normalized) associated with the red fluorescent-labeled lipid kinase isoforms, including: **a** PI4K2A, *N* = 11; **b** PI4K2B, *N* = 24; **c** PI4K3A, *N* = 21; and **d** PI4K3B, *N* = 16 and the SE (gray) for all traces. Dashed vertical line corresponds to the fusion event (0 s) of MMP9-GFP in the green channel, which was time-aligned to the red channel

Several reports suggest that the presence and activity of PI4K on synaptic vesicles or DCVs facilitates and promotes exocytosis [46–48]. Therefore, the dynamic behavior of several red-tagged lipid kinases at exocytic sites of MMP-9 was also examined, including four PI 4-kinase isoforms (e.g. PI4K2A, PI4K2B, PI4K3A, PI4K3B) [63]. Following stimulation with PMA, a reproducible accumulation of both PI4K2A and PI4K2B following the moment of exocytosis of MMP-9 was observed. With PI4K2A, the fluorescence signal remained elevated at fusion sites for at least 1 min after release (Fig. 4a), while PI4K2B, the fluorescence signal only transiently accumulated post-exocytosis of MMP-9 (Fig. 4b). Also, this brief accumulation following membrane fusion occurs consistently and reproducibly approximately five to ten seconds after the release of MMP-9. After stimulation with PMA, a rapid yet transient accumulation of PI4K3A (Fig. 4c) during membrane fusion and exocytosis of MMP-9 was observed. This dynamic was similar to that observed with the PIP^P4M^ sensor. However, no change in fluorescence around the moment of membrane fusion was observed using PI4K3B (Fig. 4d).

**Fig. 5:**
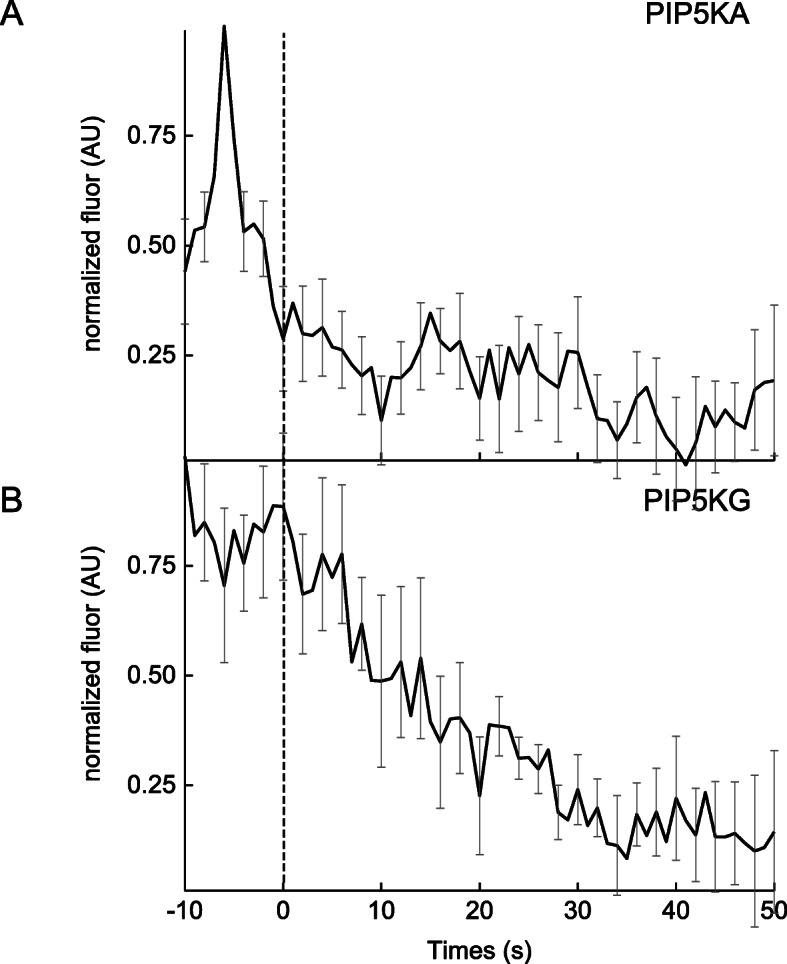
Average fluorescence intensity changes (dark line; background-subtracted and normalized) associated with the red fluorescent-labeled lipid kinase isoforms, including: **a** PIP5KA, *N* = 23; and **b** PIP5KG, *N* = 11 and the SE (gray) for all traces. Dashed vertical line corresponds to the fusion event (0 s) of MMP9-GFP in the green channel, which was time-aligned to the red channel

Next, the dynamic behavior of three different isoforms of PIP5K at exocytic sites of MMP-9 was tested. This included PIP5KA, PIP5KB, and PIP5KG, which are involved in the synthesis of PIP2. Evidence suggests a function of PIP5K in regulated exocytosis, as depletion of PIP5K from the plasma membrane caused the inhibition of DCV exocytosis and overexpression of PIP5K restored exocytosis [69]. After stimulation with PMA, various dynamic behaviors with the different red-tagged PIP5K isoforms was observed at exocytic sites of MMP-9. The PIP5KA showed decrease fluorescence over time following the exocytosis of MMP-9 (Fig. 5a). This suggests this isoform is released from sites of secretory vesicle fusion after cargo release. No exocytic events associated with MMP-9 release following cotransfection with PIP5KB were observed. This suggests a possible inhibitory effect or overexpression effect from PIP5KB on regulated exocytosis. The PIP5KG sensor exhibited the most dynamic temporal changes at exocytic sites, showing transient accumulation prior to fusion and exocytosis of MMP-9 (Fig. 5b). A rapid (and reproducible) accumulation of PIP5KG was observed approximately 5 s before the moment of membrane fusion. These results on the dynamic behavior associated with the various lipid kinases, like PI4K and PIP5K, and the lipid precursor, PI4P (or PIP), suggests these biomolecules are accumulated at sites of exocytosis of MMP-9, during or around the time of membrane fusion. Their accumulation at exocytic sites before, during, or after exocytosis of MMP-9, at fusion sites demonstrates three important points. (1) It reiterates the idea that many specialized lipids (e.g. PIP, PIP2, and DAG) are locally synthesized at exocytic sites with the help of several lipid kinases (also enriched at fusion sites). (2) It reinforces the role of lipids as organizing “elements” identifying sites of fusion and cargo release. (3) It underscores the important role that the lipid precursors of PIP2 and the upstream effectors involved in PIP2 synthesis have on regulated exocytosis in cells.

### Enrichment of protein kinase C isoforms at exocytic sites, around the moment of fusion

**Fig. 6:**
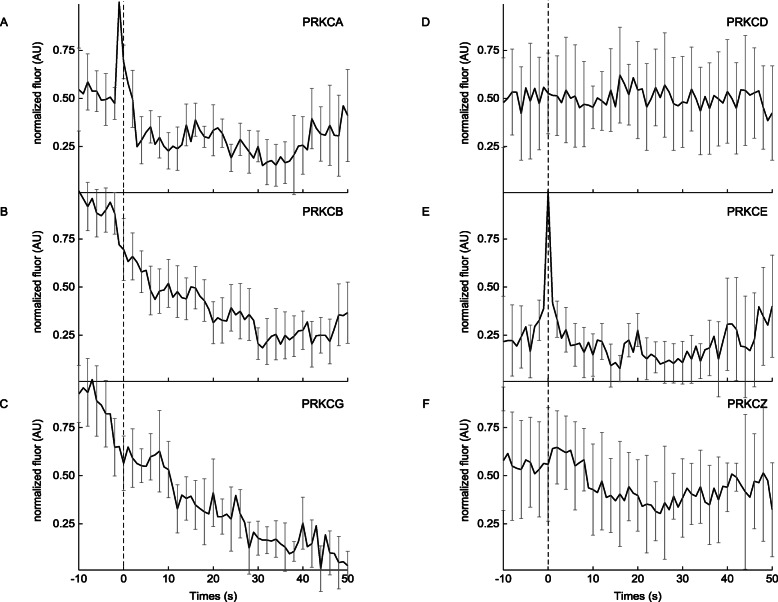
Average fluorescence intensity changes (dark line; background-subtracted and normalized) associated with the red fluorescent-labeled protein kinase C isoforms, including: **a** PRKCA, *N* = 25; **b** PRKCB, *N* = 16; **c** PRKCG, *N* = 16; **d** PRKCD, *N* = 13; **e** PRKCE, *N* = 25; and **f** PRKCZ, *N* = 5 and the SE (gray) for all traces. Dashed vertical line corresponds to the fusion event (0 s) of MMP9-GFP in the green channel, which was time-aligned to the red channel

Because of the accumulation of DAG at exocytic sites during the PMA-induced release of MMP-9, the role of PRKCs during these fusion events was examined. PRKC function is commonly examined using PMA, because it is a potent DAG analogue that acts as a PRKC activator [75–78]. Moreover, DAG stimulates exocytosis through activation of PRKCs, downstream signaling proteins involved in membrane trafficking and exocytosis [42, 71]. The temporal dynamics of the various PRKC isoforms from the three major groups was examined, including conventional (alpha, beta, and gamma), novel (delta and epsilon), and atypical (zeta), at exocytic sites of MMP-9. Following stimulation with PMA, a rapid yet transient accumulation of PRKCA and PRKCE was observed at sites of exocytosis (Fig. 6a and e). The decay kinetics associated with both isoforms show broad similarity to those observed for the DAG-sensor. While PRKCB and PRKCG do not show accumulation at the moment of fusion, both isoforms still laterally diffuse away from the exocytic site following fusion (Fig. 6b and c). Lastly, no changes in fluorescence during fusion events with the PRKCD and PRKCZ (Fig. 6d and f) were observed. Overall, this data suggests that the PRKC isoforms, PRKCA and PRKCE, are specifically accumulated at exocytic sites during membrane fusion, following PMA-induced exocytosis in MCF-7 cells. More importantly, this enrichment occurs transiently during membrane fusion and secretion of MMP-9. To further investigate the involvement of the various PRKC isoforms in the release of MMP-9, MCF-7 cells were cotransfected with MMP9-GFP and small interfering (siRNAs) against PRKCA, PRKCD, or PRKCE. Following the addition of PMA, cells transfected with siRNAs knocking down PRKCD or PRKCE caused a significant reduction in the number of exocytic events occurring on average in each cell. Cells transfected in the absence of siRNAs showed on average > ^~^ 15 fusion events total per set of cells imaged. While knockdown of PRKCD or PRKCE resulted in one to two fusion events per set. Cells transfected with siRNAs against PRKCA showed no fusion events per cell. Altogether, these results suggest that in MCF-7 cells, there are several PRKC isoforms that accumulate at exocytic sites of MMP-9 during membrane fusion.

**Fig. 7:**
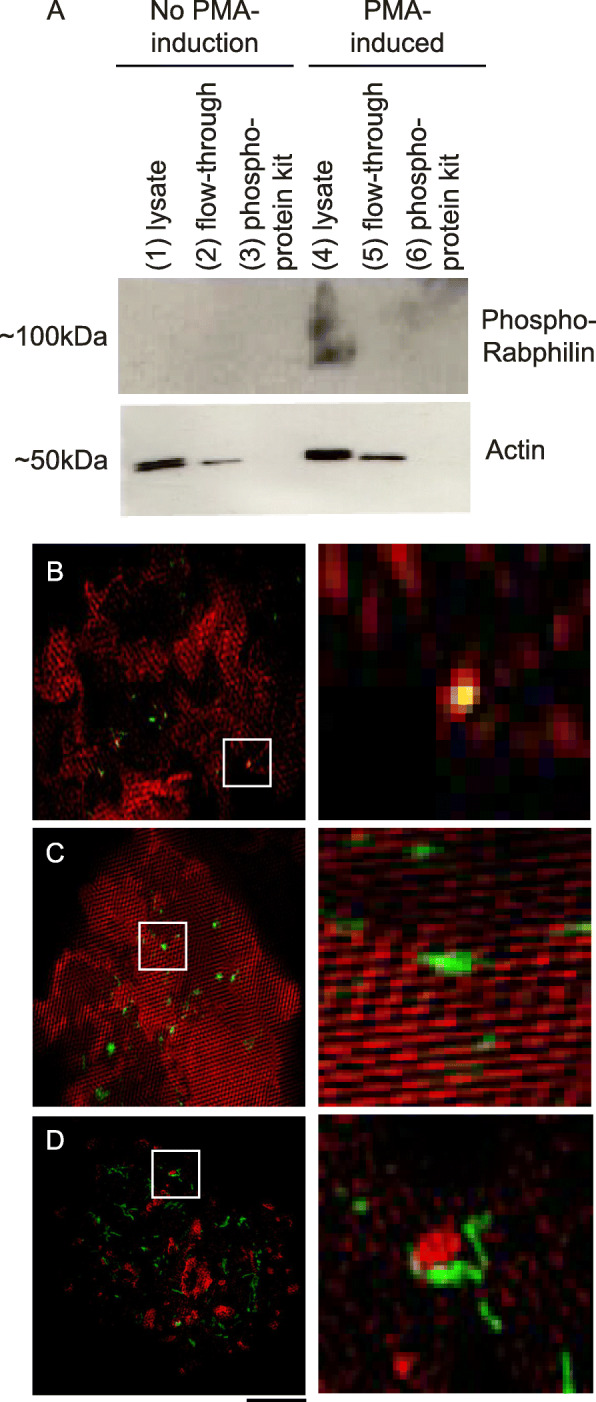
**a** Western blot, using unpurified lysates and various components, eluants versus concentrates, associated with phosphoprotein purification kit, for phospho-specific Rabphilin 3A (Ser234), without and with PMA-induction. Spatial organization, super resolution SIM images of PRKC isoforms with its potential phosphorylation targets during PMA induction. Shown on the left is the spatial arrangement of: **b**PRKCE with MMP9-GFP; **c** PRKCA with Rab27a; and **d** PRKCA with Rab27b. Shown on the right is the enlarged regions (boxes on left); same order as on the left

Since the data suggested a role of several PRKC isoforms in regulated exocytosis, a possible target of phosphorylation by the PRKCs was important to identify. PRKCs have been shown to phosphorylate several key components associated with secretory vesicles and/or exocytic machinery at fusion sites, including Rabs (and Rab effector proteins) [79, 80], vesicle bound SNARE proteins [81–83], synaptotagmins [84, 85], and Munc18 [38, 86]. Over the years few targets of phosphorylation by the PRKC proteins have been identified in the context of neuronal or neuronal-like cell lines. However, the majority of targets for regulation by phosphorylation in vivo in other secretory vesicle cell types are unknown. This has been attributed to the lack of phosphoprotein specific antibodies targeting secretory vesicle proteins or exocytic machinery in non-neuronal, MCF-7 cells. The lack of phosphoprotein specific antibodies left limited target options. However, Rabphilin 3A (Ser234) antibody was available and used to detect phosphorylation of the Rab effector protein. Rabphilin is known to have an association to exocytic machinery and is expressed in a variety of secretory vesicle cell types, including both neuronal and non-neuronal cells lines. Lysates obtained from cells that had been either PMA-uninduced or PMA-induced were purified using a phosphoprotein purification kit. Then western blot analysis was conducted using an anti-phospho Rabphilin 3A (Ser234) antibody, which is specific for phosphorylated Rabphilin. No enrichment of phosphorylated Rabphilin following purification using the phosphoprotein purification kit was observed. This is likely because the purification results in large loss of proteins and subsequent lack of detection using the kit (Fig. 7a). However, the western blots did show phosphorylated Rabphilin following PMA-induction in unpurified lysates. While blots using unpurified lysates from uninduced cells revealed no visible band corresponding to phosphorylated Rabphilin 3A (Fig. 7a). This result suggests that PRKC proteins possibly target Rab effectors proteins, which is consistent with previous studies showing different Rab proteins as targets for PRKC isozymes [80, 87–89].

It was hypothesized that any target of the PRKCs involved in exocytosis would require close proximity between PRKCs and secretory vesicle proteins at sites of PMA-induced exocytosis. Therefore, the super-resolution technique, TIRF-SIM (total internal refection fluorescence structured illumination microscopy) was used to determine the proximity (i.e. spatial positioning) of PRKCs to secretory vesicles containing MMP-9, following PMA-induction. TIRF-SIM has recently been used for high resolution imagining, improved spatial resolution, and enhanced resolution from live cell imaging. Specifically, this technique has be used to detect a variety of colocalizations and dynamic interactions between organelles or with the plasma membrane [90–92]. Vesicles containing MMP-9 colocalized strongly with the PRKCE isoforms (Fig. 7b) were observed following PMA stimulation. However, no direct colocalization with PRKCA or PRKCG following induction with PMA was observed (data not shown). Also, the spatial positioning of PRKC isoforms was examined with Rab27 GTPase isoforms (organizing element #1; specialized scaffolding proteins), which revealed punctate localization and a close proximity of PRKCA and Rab27b (Fig. 7d), following PMA induction. There was no punctate localization or close proximity associated with PRKCA and Rab27a (Fig. 7c) with any isoform. No direct localization with Rab27b and PRKCG or PRKCE was observed (data not shown).

### No accumulation of phosphatic acid at exocytic sites around the moment of fusion

Various other lipids and their associated protein effectors have been implicated in regulated exocytosis, including phosphatidic acid, PA [44, 51]. PA recruits proteins for vesicle fusion or priming events (such as SNAREs) and facilitates efficient vesicle exocytosis [35–37]. Given the evidence of regulatory lipid turnover at exocytic sites, the role of PA at exocytic sites was examined. Because DAG is further metabolized to PA, via the activity of diacylglycerol kinase (DGK), the dynamics of DGKs and PA at fusion sites was monitored during PMA-induced exocytosis of MMP-9. A small number of fusion events was observed following overexpression of the PA-sensor and there was no change in fluorescence associated with the PA-sensor at exocytic sites during membrane fusion (Supplemental Figure 2A). Moreover, imaging the three different isoforms of DGK (alpha, delta, and gamma), only a few fusion events and no evidence of these proteins accumulating at sites of exocytosis during the moment of fusion was observed (Supplemental Figure 2B-D). This suggests that PA and associated effector proteins, DGKs, display little temporal dynamic changes during exocytosis of MMP-9 at individual sites of fusion in MCF-7 cells. It is important to note that overexpression of the majority of the lipid sensors and effector proteins did not have an effect on the number of fusion events (*N*; see figure legends) examined per set. However, unlike the others, the PA-sensor and the DGK’s all showed reduced number of fusion events.

### Prenylation mutations (a lipid modifications) of Rab27 isoforms alter MMP-9 release dynamics at exocytic sites

**Fig. 8:**
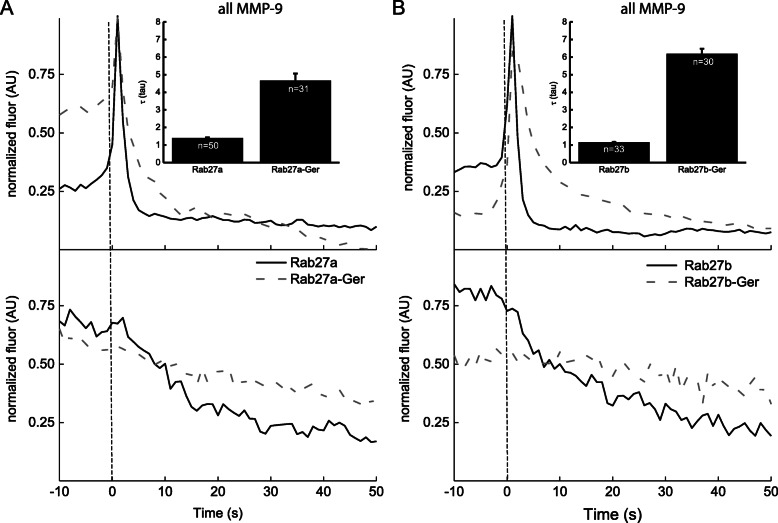
**a** Average fluorescence intensity changes (dark line; background-subtracted and normalized) associated with MMP9-GFP (green channel; top), co-expressed with Rab27a-WT, wild-type (solid black) or Rab27a-Ger (dashed gray) in the red channel (bottom). **b** Average fluorescence intensity changes (dark line; background-subtracted and normalized) associated with MMP9-GFP (green channel; top), co-expressed with Rab27b-WT (solid black) or Rab27b-Ger (dashed gray) in the red channel (bottom). Dashed vertical line corresponds to the fusion event (0 s) of MMP9-GFP in the green channel, which was time-aligned to the red channel

Also, the role of prenylation of Rab27 isoforms at sites of exocytosis of MMP-9 was examined. Protein prenylation is a specific type of lipid modification made to some proteins, including Rab GTPases and involves the transfer of either geranylgeranyl or a farnesyl moiety to C-terminal cysteines on target proteins [93]. Geranylgeranyl modifications are important in mediating protein-protein interactions and protein-membrane interactions [94] and both Rab27 isoforms undergo geranylgeranylation [93]. It was previously determined that both Rab27a and Rab27b, which are involved in the late stages of exocytosis, were localized to sites of exocytosis of MMP-9 and potentially act as scaffolding proteins (another type of organizing “element”) at exocytic sites [57]. Mutations made to the geranylgeranyl region of both Rab27 also delayed or slowed decay kinetics associated with MMP-9 exocytosis. Fusion decay kinetics (green channel) associated with Rab27a mutants increased by approximately four-fold from in τ = 1.3 ± 0.0 s (WT) to τ = 4.7 ± 0.4 s (prenylation) (Fig. 8a; top), while Rab27b prenylation mutants increased by approximately six-fold, from τ = 1.1 ± 0.0 s (WT) to τ = 6.2 ± 0.3 s (prenylation) (Fig. 8b; top). Examination of the protein dynamics in the red channel with the Rab27a- and Rab27b-specific prenylation mutants at these exocytic sites of MMP-9 showed slower dissociation from these sites. Similar to that observed with their WTs, overexpression of Rab27a- and Rab27b-specific prenylation mutants still displayed localization to exocytic sites prior to fusion that subsequently diffused away from these sites following fusion (Fig. 8a and b; bottom). The major difference occurs post-fusion, where the Rab27a- and Rab27b-specific prenylation mutations (Rab27a-Ger or Rab27b-Ger) are lost from sites of fusion slower than their WT counterparts. These results in both the green and red channels are consistent with the data reported with the constitutively active or dominant-negative versions of these Rab27 mutants previously reported [57]. More importantly, the temporal dynamics associated with the Rab27 isoforms (organizing “element” #1: scaffolding proteins) contrast significantly with the overall dynamic behavior associated with several specialized lipids (organizing “element”#2: lipids).

## Discussion

The process of regulated exocytosis has been a topic of research for several decades and has led to the discovery of a host of different proteins and lipids involved throughout this process. The regulatory lipid, PIP2, is the best example of the involvement of a lipid in regulated exocytosis, with roles in membrane fusion associated with regulated DCV exocytosis in neuroendocrine cells, synaptic vesicles exocytosis in neurons, and constitutive vesicle exocytosis in a variety of cell types [50]. There is growing evidence suggesting that PIP2 acts as a key organizing “element”, enriched in microdomains of the plasma membrane, spatially defining the position of exocytic sites and temporally identifying the precise timing of membrane fusion events [23, 49, 50]. This spatial and temporal organization is presumably accomplished through the local synthesis and degradation of PIP2 at these spatiotemporally defined sites of membrane fusion.

**Fig. 9:**
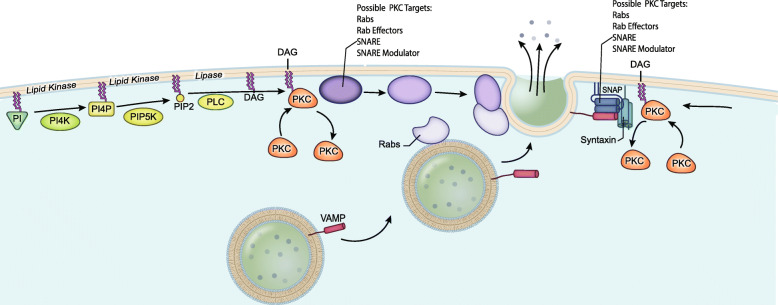
A depiction of the PIP2-mediated signaling pathway on the regulated exocytosis of MMP-9 in MCF-7 cells. Illustration shows a step associated with dynamic cascade of PIP2 formation and subsequent breakdown, and the lipid kinases and protein kinases associated with exocytic sites

Here, the spatiotemporal organization and dynamics of specialized lipids surrounding the PIP2-mediated signaling pathway at fusing secretory vesicles containing the pro-tumor marker, MMP-9 was investigated in living MCF-7 breast cancer cells. TIRF was used to probe the lipid environment and effectors proteins associated with these lipids to better define their spatial patterning and temporal dynamic behavior, during membrane fusion at exocytic sites of MMP-9. To this end, systematic spatial mapping and temporal dynamics of key specialized lipids and proteins implicated in the PIP2-mediated signaling pathway were determined at individual exocytic sites of MMP-9. The major synthetic pathway for the formation of PIP2 (i.e. PIP2-mediated signaling pathway; Fig. 9) utilized by cells, involves a cascade of lipids. These specialized lipids are spatially and temporally regulated through the actions of kinases, phosphatases, and phospholipase, which are locally synthesized or accumulated at exocytic sites of MMP-9 [23, 26, 44, 49–51].

Consistent with this idea, the dynamic behavior of several lipids observed using two-color TIRF revealed an accumulation of: several lipids, (e.g. PIP, PIP2, and DAG); some lipid kinase isoforms responsible for their synthesis (e.g. PI4K2B, PI4K3A, and PIP5KA); and several downstream protein kinase isoform effectors (e.g. PRKCA and PRKCE) at exocytic sites. From the data, one general dynamic behavior (i.e. transient accumulation around the time of membrane fusion) of lipid-sensors, lipid kinases, and protein kinases (red channel) was identified at sites of exocytosis of MMP-9 (green channel). Spatial patterning and temporal dynamic measurements suggest that these molecules are not highly colocalized or enriched at docked secretory vesicles containing MMP-9 in the absence of PMA. However, following stimulation with PMA, which triggers regulated exocytosis, many of these molecules showed rapid and transient accumulation at exocytic sites. This accumulation centered around the moment of cargo release and was followed by a slow decay.

After stimulation with PMA, an enrichment of the lipid, PI4P, was observed using the PIP^P4M^ lipid sensor (Fig. 3a) and two isoforms of the PI-specific lipid kinase, PI4K, using PI4K2B and PI4K3A (Fig. 4b and c), at exocytic sites around the time of membrane fusion. For the four PI4K variants tested (Fig. 4a-d), PI4K2A isoform showed prolonged enrichment at fusion sites during and after exocytosis, while the PI4K2B isoform showed transient enrichment after exocytosis. However, the PI4K3A isoform showed a rapid yet transient accumulation centered on the moment of membrane fusion at exocytic sites. While the PI4K3B isoform showed no change in fluorescence signal before, during, or after membrane fusion events. The next sequential step in PIP2 synthesis is the conversion of PI4P (or PIP) into PIP2 by the PI-specific kinase, PIP5K. A rapid and transient accumulation of the regulatory lipid, PIP2 (Fig. 2d) was observed during exocytosis of MMP-9 and accumulation of the PIP5KA isoform (Fig. 5a) prior to exocytosis of MMP-9. Collectively, this data points to a potential in vivo spatial and temporal role of the lipids and PI-specific lipid kinases at exocytic sites and the release of the secretory vesicle cargo, MMP-9. It is speculated that the PI-derived lipids, PIP and PIP2 are locally synthesized at fusion sites during exocytosis of MMP-9. Moreover, the accumulation of PIP and PIP2 is accomplished through the local accumulation, at exocytic sites around the moment of fusion, of the lipid kinases isoforms, PI4K2B, PI4K3A, and PIP5KA, responsible for their synthesis. Overall, taken together, the dynamics associated with the various PI4K and PI5PK isoforms implies a distinct spatial and temporal organization at sites of exocytosis of MMP-9 in MCF-7 cells.

In the PIP2-mediated signaling pathway (Fig. 9), PIP2 levels can be regulated by the PI-specific lipid kinases that lead to PIP2 synthesis. However, PIP2 concentrations can also be regulated by the degradation of PIP2 by PLC, into DAG, which acts as a secondary messenger to activate the downstream effectors: (1) lipid kinase family, DGKs; or (2), protein kinase family, PRKCs. A transient accumulation of DAG (Fig. 2h) was observed during exocytosis of MMP-9, with kinetics broadly similar to that observed with PIP2. As it relates to DGK, a downstream effector of DAG, there was no dynamic fluorescence changes associated with DGK isoforms or with the PA-lipid sensor, following DGK activity on DAG (Supplemental Figure 2A-D). While for PRKC, the other downstream effector of DAG, a rapid yet transient accumulation of several of the PRKC isoforms (PRKCA and PRKCE) was observed during the exact moment of fusion at exocytic sites of MMP-9 (Fig. 6a and e). This is similar to that detected for the DAG lipid sensor and again suggests possible isoform specific spatial and temporal roles of the various PRKCs at exocytic sites during membrane fusion. It is hypothesized that DAG acts at sites of exocytosis of MMP-9, to possibly recruit and activate PRKCs to potentially phosphorylate exocytic proteins at fusion sites.

In neuroendocrine, PC12 cells, PRKC phosphorylates several exocytic proteins, including Munc18, SNAP25, and synaptotagmin [83, 86, 95, 96]. Furthermore, this phosphorylation facilitates regulated exocytosis in these cells by modulating membrane-attached exocytic machinery [97]. A similar scenario could exist in MCF-7 cells, where PRKC is locally recruited to exocytic sites, during the moment of membrane fusion. This would allow the PRKCs to phosphorylate a variety of proteins associated with fusogenic secretory vesicles containing MMP-9. Although, multiple targets of phosphorylation by PRKC could not be determined, the blots suggest that Rabphilin, a Rab effector protein, is a potential target (Fig. 7). Moreover, using super-resolution TIRF-SIM showed that after stimulation with PMA, there is close proximity (i.e. colocalization) between PRKCE and secretory vesicles containing MMP-9 (Fig. 7). Overall, the data showed isoform-specific PRKC locally accumulated at exocytic sites of MMP-9 and suggests a possible spatial and temporal role of PRKC at exocytic sites in MCF-7 cells.

The TIRF-based imaging data presented here showed that the PIP2-mediated signaling pathway is involved in the spatiotemporal organization during membrane fusion, at exocytic sites of MMP-9 in MCF-7 cells. More importantly, this cascade of lipids in the pathway may serve as organizing “elements”, spatially and temporally coordinating regulated exocytosis in these breast cancer cells. Systematic mapping was done to determine the dynamic behavior involved in the lipid signaling cascade from PI to PRKC. The lipids, which include PIP, PIP2, and DAG, are regulated at the level of synthesis and degradation, through the interconversion by specific kinases and phospholipases. This interconversion locally controls the concentrations of these lipids at sites of exocytosis. These results showed that the lipids in the PIP2 signaling pathway are locally synthesized or recruited to exocytic sites of MMP-9 in MCF-7 cells. At precisely the moment of fusion, their transient accumulation is also accompanied by transient accumulation of the enzymes involved in their synthesis or their downstream effectors, including lipid kinases and protein kinases (i.e. PRKCs), respectively. The low spatial patterning or low colocalization, as well as, the temporal dynamic behavior (i.e. rapid yet transient accumulation) associated with the specialized lipids (i.e. organizing “element” #2) at exocytic sites contrasts drastically with the specialized scaffolding proteins (organizing “element” #1). With these specialized scaffolding proteins, high colocalization and decisively different temporal dynamics associated with the Rab proteins (e.g. Rab27a and Rab27b, Fig. 8; bottom), Rab effector proteins (e.g. Rabphilin), and SNARE proteins (e.g. VAMP3) were observed at these same exocytic sites in MCF-7 cells [57]. Specifically, these scaffolding proteins stably associate with docked secretory vesicles before exocytosis and are subsequently lost slowly from exocytic sites at the moment of fusion (via lateral diffusion in the plasma membrane) [57].

### Study strengths and limitations

These results reiterate the utility and broad applicability of this two-color TIRF imaging-based approach for systematically mapping the molecular composition, spatial organization, and dynamic temporal behavior of discrete cellular processes like that surrounding regulated exocytosis [55–57, 67]. It is important to note that all of the spatiotemporal information acquired here using this TIRF method implies only correlative associations. Specifically, the recruitment of lipid sensors and tagged kinase enzymes (i.e. lipid kinases and protein kinases) involved in PIP2-mediated signaling pathway to exocytic sites is correlated with the exocytosis of MMP-9 from secretory vesicles in MCF-7 cells and does not prove a functional or mechanistic connection with exocytosis. The goal of this imaging-based TIRF method was to establish the spatial organization and temporal dynamics of specialized lipids (organizing element #2) associated with membrane fusion, at exocytic sites of MMP-9 in MCF-7 cells. This study is meant to complement, not replace, traditional functional studies used to probe the spatiotemporal organization associated with these exocytic sites. Future biochemical studies will be essential in directly uncovering the functional associations of these specialized lipids (organizing element #2) at sites of regulated exocytosis of the pro-tumor marker, MMP-9 in MCF-7 breast cancer cells and could ultimately further the understanding of cancer progression and metastasis.

There are limitations associated with this TIRF-based imaging screen that should be considered with this work. The imaging screen used here relies on overexpression, which can cause problems, since protein localization, dynamics, or other cellular behaviors could be affected by varying protein levels. Therefore, it cannot be completely excluded that the spatiotemporal organization observed here could be altered as a result of the behavior associated with overexpression of the fluorescently-tagged lipid sensors or effector proteins. Furthermore, overexpression experiments (like ours) should be considered as qualitative experiments designed to identify potential novel interactions and mechanisms for further study. The in vivo study in MCF-7 cells has helped identify potential targets, which can be examined more quantitatively both, in vitro and in vivo in different cancer cells types. This will be especially important when examining spatiotemporal organization (*i.e* the three organizing “elements”) that inevitably exist in vivo in different cancer cell lines.

## Conclusions

The current understanding of the spatial organization and temporal dynamics associated with the three distinct categories of organizing “elements” during regulated exocytosis has come extensively from a limited number of categories of secretory cell types. There are four major categories of cells capable of undergoing regulated exocytosis, including neuronal, endocrine, exocrine, and hematopeoietic cells [98]. However, an overwhelming majority of the information known about regulated exocytosis comes from either neuronal or endocrine cells types, using a limited number of secretory vesicle markers specific to those cell types. Therefore, the discovery that MMP9-GFP could be used to image regulated exocytosis in the exocrine, MCF-7 cells line, allows the spatial organization and temporal dynamics associated with protein and lipid involvement in regulated exocytosis in other secretory cell types to be understood [57]. The TIRF-based imaging system was used to systematically determine the spatial organization and temporal dynamics of lipids (and effector proteins) associated with membrane fusion and exocytosis of MMP-9 in MCF-7 cells. The local accumulation of the PIP2-mediated signaling lipids, like PIP, PIP2, and DAG at exocytic sites of MMP-9, during PMA-stimulated exocytosis in MCF-7 cells, establishes local enrichment or accumulation of these specialized lipids to exocytic sites. These specialized lipids can facilitate possible protein-lipid interactions needed at exocytic sites and provide possible protein-recruiting mechanisms that offer additional supplementary levels of control of the exocytic machinery in the cell.

## Supplementary information

**Additional file 1: Figure S1.** Average fluorescence intensity changes (dark line; background-subtracted and normalized) associated with the red fluorescent-labeled: (A) farneslyated-mCherry protein, *N* = 34; (B) mCherry protein, *N* = 30; and (C) mutated PIP2-sensor, *N* = 17 and the SE (gray) for all traces. Dashed vertical line corresponds to the fusion event (0 s) of MMP9-GFP in the green channel, which was time-aligned to the red channel. **Figure S2.** Average fluorescence intensity changes (dark line; background-subtracted and normalized) associated with the red fluorescent-labeled (A) PA-sensor, *N* = 3; (B) DGKA, *N* = 6; (C) DGKD, *N* = 3; (D) DGKG, *N* = 4 and the SE (gray) for all traces. Dashed vertical line corresponds to the fusion event (0 s) of MMP9-GFP in the green channel, which was time-aligned to the red channel.

## Data Availability

The datasets used and/or analyzed during the current study are available from the corresponding author on reasonable request.

## Abbreviations

MMP-9: Matrix metalloproteinase-9
SNAREs: Soluble N-ethylmaleimide–sensitive factor attachment protein receptors
PIP: Phosphatidylinositol 4-phosphate
PIP2: Phosphatidylinositol-4,5-biphosphate
DAG: Diacylglycerol
DGK: Diacylglycerol kinase
PMA: Phorbol myristate acetate
PI4K: Phosphatidylinositol 4-kinase
PIP5K: Phosphatidylinositol 4-phosphate 5-kinase
PRKC: Protein kinase C
