# Supplementary material for: Imaging the rapid yet transient accumulation of regulatory lipids, lipid kinases, and protein kinases during membrane fusion, at sites of exocytosis of MMP-9 in MCF-7 cells
Source: Lipids Health Dis. 2020 Aug 23;19:195. doi: 10.1186/s12944-020-01374-9 (PMC7444259; doi:10.1186/s12944-020-01374-9)
Supplement: Supplementary file 1 — Additional file 1: Figure S1. Average fluorescence intensity changes (dark line; background-subtracted and normalized) associated with the red fluorescent-labeled: (A) farneslyated-mCherry protein, N = 34; (B) mCherry protein, N = 30; and (C) mutated PIP2-sensor, N = 17 and the SE (gray) for all traces. Dashed vertical line corresponds to the fusion event (0 s) of MMP9-GFP in the green channel, which was time-aligned to the red channel. Figure S2. Average fluorescence intensity changes (dark line; background-subtracted and normalized) associated with the red fluorescent-labeled (A) PA-sensor, N = 3; (B) DGKA, N = 6; (C) DGKD, N = 3; (D) DGKG, N = 4 and the SE (gray) for all traces. Dashed vertical line corresponds to the fusion event (0 s) of MMP9-GFP in the green channel, which was time-aligned to the red channel. [file 12944_2020_1374_MOESM1_ESM.pdf]

SFigure 1

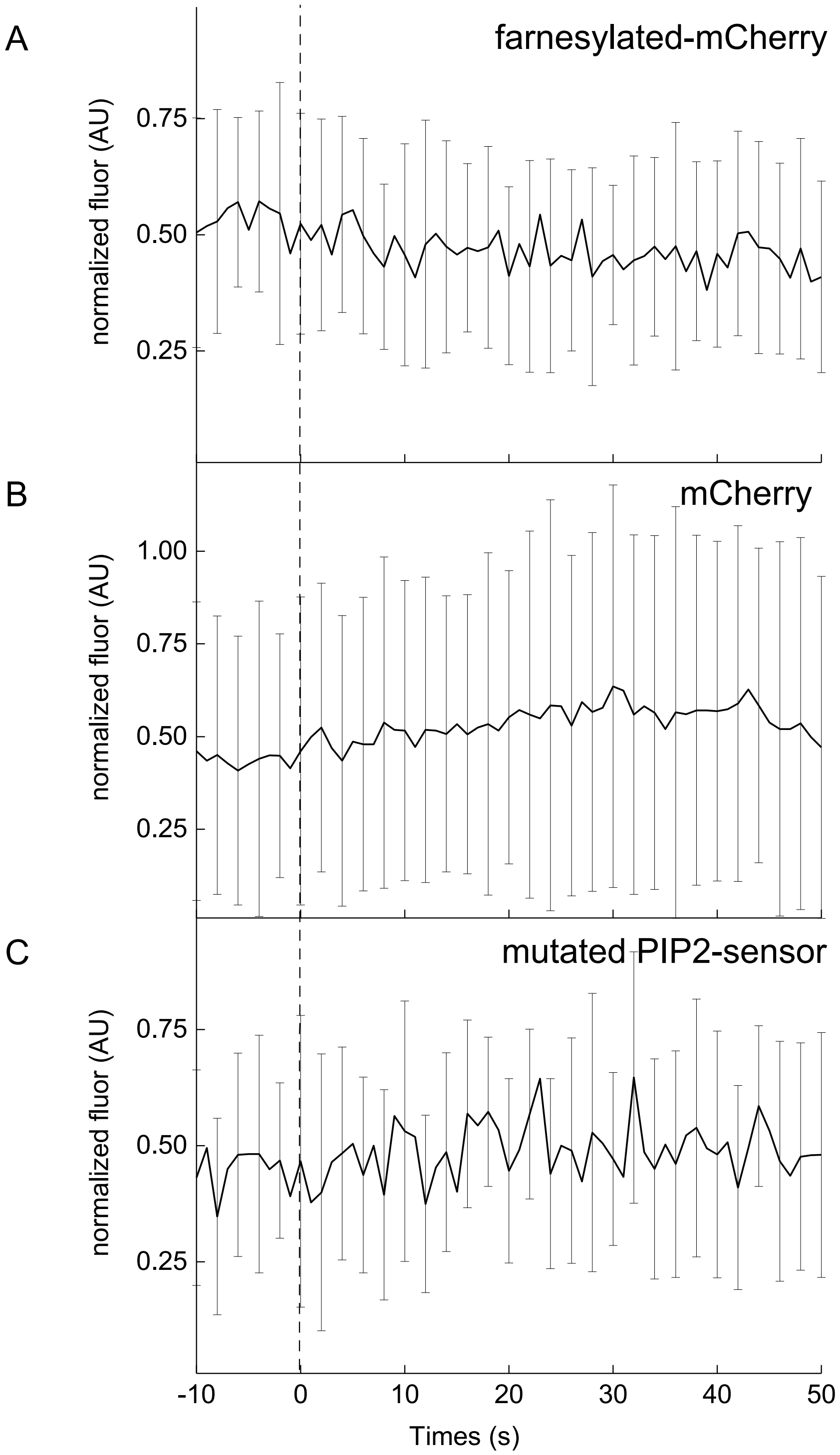

Supplemental Figure 1: Average fluorescence intensity changes (dark line; background-subtracted and normalized) associated with the red fluorescent-labeled: (A) farneslyated-mCherry protein, N=34; (B) mCherry protein, N=30; and (C) mutated PIP2-sensor, N=17 and the SE (gray) for all traces. Dashed vertical line corresponds to the fusion event (0 seconds) of MMP9-GFP in the green channel, which was time-aligned to the red channel.

SFigure 2

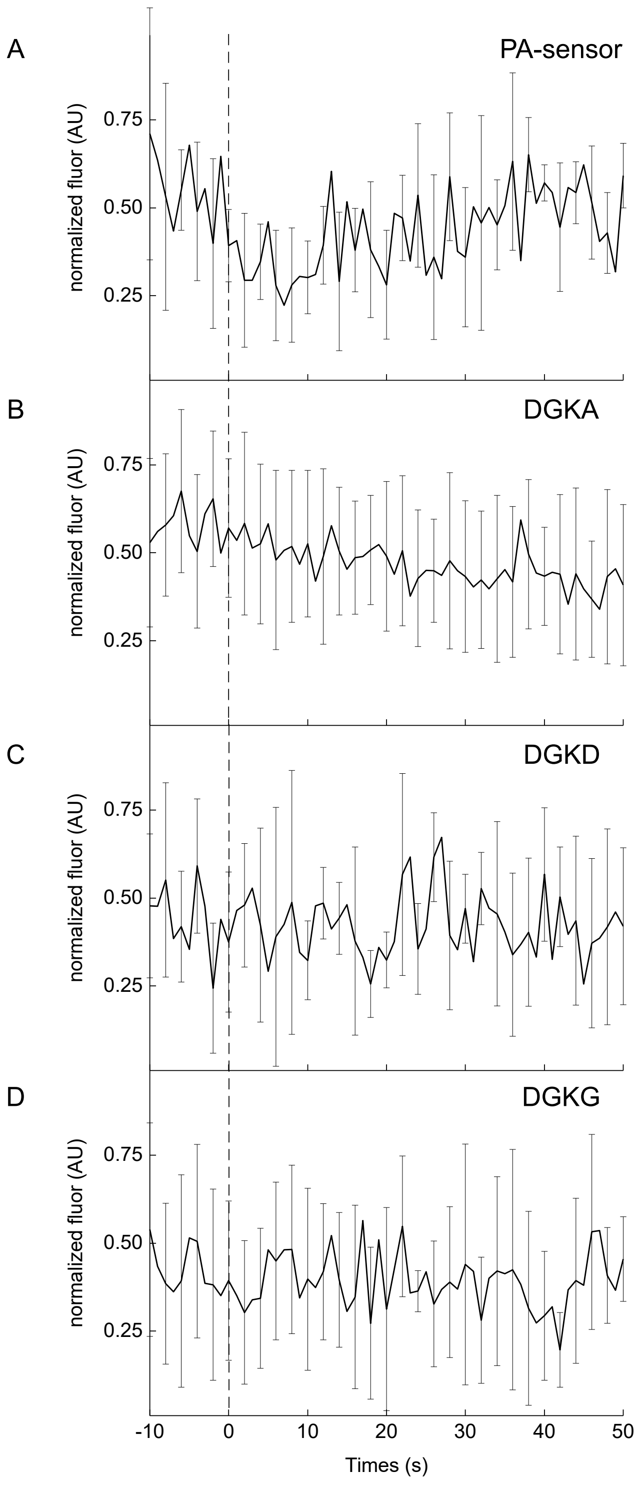

Supplemental Figure 2: Average fluorescence intensity changes (dark line; background-subtracted and normalized) associated with the red fluorescent-labeled (A) PA-sensor, N=3; (B) DGKA, N=6; (C) DGKD, N=3; (D) DGKG, N=4 and the SE (gray) for all traces. Dashed vertical line corresponds to the fusion event (0 seconds) of MMP9-GFP in the green channel, which was time-aligned to the red channel.
